# Anterior Versus Posterior Stabilization of Subaxial Cervical Spine Fracture-Dislocations, Dislocations and Subluxations: A Retrospective Cohort Study of Neurological and Radiological Outcomes

**DOI:** 10.3390/jpm16070348

**Published:** 2026-06-26

**Authors:** Gorazd Kovac, Ernst Josef Müller, Martin Liebhauser, Jochen Jung, Haro Stettner, Martin Halbherr

**Affiliations:** Department of Trauma Surgery, Klinikum Klagenfurt am Wörthersee, 9020 Klagenfurt, Austria; gorazd.kovac@kabeg.at (G.K.);

**Keywords:** cervical spine injuries, spinal fractures, intervertebral disc displacement, spinal cord injuries, treatment outcome, retrospective studies

## Abstract

**Background**: Dislocations and fracture-dislocations of the lower cervical spine represent complex injuries with a high risk of neurological damage. Especially in the presence of a confirmed traumatic disc lesion, an anterior surgical approach is described as favoured in the literature. However, studies show that with sufficient reduction technique, even in the presence of a confirmed disc protrusion, posterior stabilization can be considered a safe therapeutic option. The aim of this study is to analyze anterior and posterior treatment of dislocations and fracture-dislocations of the subaxial cervical spine with regard to neurological and radiological outcomes. **Methods**: In our monocentric cohort study, we investigated the immediate postoperative radiological and neurological outcome depending on the chosen surgical approach and the presence of a disc protrusion. Patients treated at our centre between January 2005 and June 2025 were included. Patients with preoperative complete spinal cord injury were excluded. Neurological status was assessed using the ASIA score preoperatively at admission and postoperatively at discharge or prior to staged surgery. **Results**: A total of 92 patients were included in the study. Most patients showed an ASIA score C (33.7%). A total of 49 patients (53.3%) were operated anteriorly and 42 patients (45.6%) posteriorly. One patient was primarily stabilized bilaterally. Nine patients initially treated anteriorly had to be secondarily stabilized additionally from posterior. In both groups, neurological deterioration occurred in one case. All other patients remained stable on the ASIA score or improved by at least one point on the scale. **Conclusions**: The findings provide evidence in favour of a personalized, pathology-oriented approach to lower cervical spine fracture-dislocations rather than selecting the surgical approach based solely on the presence of traumatic disc protrusion. Further prospective studies are needed to validate these observations.

## 1. Introduction

Injuries of the lower cervical spine are among the most severe and complex traumas. Particularly in dislocations, fracture-dislocations, and facet-associated subluxations, there is a high risk of neurological complications up to complete spinal cord injury. These injuries are rare but associated with high morbidity, mortality and a massive socioeconomic burden [1,2].

While injuries of the upper cervical spine are more commonly associated with low-energy trauma and therefore occur in older individuals, injuries of the lower cervical spine are more frequently associated with high-energy trauma and are thus typical injuries of young male patients [3]. The most frequent localization of injury in the context of high-energy trauma is described at C6/C7 [4].

Particular importance is attributed to the question of the optimal timing and the correct type of reduction. The time from occurrence of the injury to adequate reduction is known as a critical factor and has already been described. In a study by Grant et al., early closed reduction, even without MRI, was advocated in awake and neurologically assessable patients, especially in the presence of an incomplete spinal cord syndrome [5]. Another study underlines these findings and concludes that the success of reduction and neurological recovery in cervical spine dislocations depend significantly on the shortest possible time interval between trauma and primary reduction [6]. Publications as well as large meta-analyses advocate a favourable outcome when surgery is performed within 24 h after trauma [7,8].

In contrast, there is concern about iatrogenic spinal cord compression, particularly in the presence of traumatic disc herniation [9]. The literature shows that in approximately 30–40% of patients with fracture-dislocations of the lower cervical spine, a disc protrusion is observed [10,11].

Both anterior and posterior surgical approaches are established, safe, and effective procedures for the surgical treatment of dislocations and subluxations of the lower cervical spine. A clear advantage of a specific approach has not yet been demonstrated. Therefore, the decision for the respective surgical approach should be made individually and should be based on the respective injury configuration, the presence of concomitant injuries, the neurological findings, and the available surgical resources.

The exclusively anterior surgical approach has been established in recent years as a safe and effective procedure. The main advantages include direct anterior decompression, the possibility of removing discogenic material, reconstruction of sagittal alignment, and stabilization using plate osteosynthesis. Compared to posterior or combined approaches, soft tissue damage is lower, and reduced blood loss and shorter operative times as well as hospitalization times are observed, although associated with an overall higher complication rate [12,13,14].

A posterior or combined surgical approach is recommended particularly in situations where sufficient reduction cannot be achieved via an exclusively anterior approach, especially in chronic dislocations, complex fracture patterns, or pronounced facet interposition. Furthermore, a posterior approach may be required in cases of significant posterior soft tissue damage or after previous anterior surgery. Long-term studies indicate that both approaches achieve comparable neurological and functional outcomes. While the anterior approach tends to be associated with better preservation of cervical lordosis, some studies report a lower complication rate for the posterior approach [14].

A Japanese research group led by Nakashima describes that posterior stabilization, even in the presence of confirmed disc protrusion, does not represent a fundamental contraindication if reduction is adequately performed. Another Korean study supports the posterior approach and indicates that disc removal can be performed posterolaterally if necessary [15,16].

The aim of this study was to compare anterior and posterior stabilization of subaxial cervical fracture-dislocations, dislocations and subluxations with regard to neurological and radiological outcomes, with attention to the influence of traumatic disc protrusion.

## 2. Materials and Methods

### 2.1. Study Design

For our retrospective cohort analysis, patients were included who were surgically treated at our centre between 1 January 2005 and 30 June 2025 for unstable injuries of the lower cervical spine (disc C2/3—disc C7/T1), defined as facet dislocation, subluxation, dislocation, or fracture (-dislocation). Further inclusion criteria were the presence of a pre- and postoperative neurological status (ASIA score), as well as preoperative imaging by means of CT and/or MRI. During the study period, neurological status was increasingly assessed using the ASIA score rather than the Frankel grading system, reflecting the gradual adoption of the more detailed and standardized ASIA classification. To ensure consistency across the cohort, Frankel grades were mapped to their corresponding ASIA categories A–E for the purposes of the present analysis.

A total of 92 patients were included in the study after those with a complete spinal cord injury (*n* = 28) were initially excluded. The recruitment of the study population was carried out via a structured evaluation of the hospital internal documentation system (Orbis, PACS, surgical documentation). The search was performed systematically based on ICD-10 diagnosis codes, existing admission diagnoses according to internal documentation, and available surgical reports. Based on the available imaging, the type of injury was reassessed.

The patients were divided into two groups, depending on whether surgical stabilization was performed primarily anteriorly or primarily posteriorly. Assignment to the respective intervention group (anterior vs. posterior) was based on the first operation performed according to the surgical report. If a combined or staged treatment was performed, the primary approach strategy was still used as the grouping criterion.

Patients assigned to group A were primarily decompressed via an anterior approach and subsequently stabilized by plate osteosynthesis after insertion of an autologous bone graft. Patients in group B underwent posterior instrumentation using a screw-rod system after reduction.

The study was approved by the institutional ethics committee of the centre (Ethics Committee Carinthia; EK number S2025-15). All methods were carried out in accordance with the relevant local and national guidelines and regulations.

### 2.2. Endpoints

The primary endpoint of the study is the change in ASIA score preoperatively at the time of admission and postoperatively at the earliest possible time of neurological assessment depending on the chosen surgical strategy and the MRI-verified presence of a disc protrusion. Analyses regarding traumatic disc protrusion were restricted to patients with available MRI examinations. The MRI was evaluated by a radiologist and a trauma surgeon specialized in spine surgery.

Secondary endpoints examine treatment stability with regard to radiological criteria depending on the chosen surgical approach, as well as the necessity of secondary interventions due to postoperative radiologically proven instability and neurological deterioration in the ASIA score. Radiological instability was defined as an increase in anterior and posterior translation > 4 mm, segmental kyphosis > 11°, or facet uncovering > 50% [17]. The timing of the first postoperative radiological assessment was chosen at the time of suture removal about 2 weeks after surgery, as this allows improved imaging evaluation after initial wound healing and supports early detection of postoperative complications. In the absence of further follow-up (e.g., early transfer to an external hospital), no comparable data could be obtained. In the majority of patients, the follow-up period for further control of radiological and neurological course assessment extended over at least several months. Neurological or radiological deterioration was defined as a primary complication. Secondary complications included surgery-specific complications such as superficial or deep wound infection, respiratory complications (pneumonia, etc.), implant failure/malposition of screws/plates, implant breakage, hoarseness, or postoperative dysphagia.

### 2.3. Statistical Methods

All analyses were performed using the programming language R (version 4.0.3, 2020) and (elementary statistics and descriptive parameter calculation) in HP-RPL (version 2.15, 2009; Hewlett-Packard Company, San Diego, CA, USA). Parametric and non-parametric standard procedures were used to calculate mean, standard deviation (SDev), standard error of the mean (SEM), and median (Med). Binomial tests were used for comparisons of proportions. Fisher-Yates test for analyses of 2 × 2 tables and frequency comparisons were used alongside the Jonckheere test for comparison of value lists and trend analysis.

## 3. Results

A total of 92 patients, 74 (80.4%) men and 18 women (19.6%) with a mean age of 55.8 years were included in the study. The youngest patient was 15 years old, and the oldest included patient was 88 years old at the time of injury. The most frequently observed injury type was unilateral locked dislocation with fracture of one articular process (*n* = 26, 28.3%), followed by unilateral perched dislocation with fracture of one articular process (*n* = 17, 18.5%) and bilateral perched dislocation (*n* = 13, 14.1%). At the time of the initial assessment of preoperative neurological status, 31 patients (33.7%) had an ASIA score C. Exactly 23 patients (25.5%) showed a radicular deficit at hospitalization; 20 patients (21.7%) were admitted with an ASIA score E. A total of 13 patients (14.1%) were initially classified as ASIA D. Only 3 patients (3.3%) were classified as ASIA B [Table 1].

The most frequent injury level was found in the segments C6/7 (52.2%), followed by C5/C6 (30.4%) and C4/C5 (7.6%) [Table 2]. An inversely proportional relationship between injury level and age was observed with a highly significant trend toward decreasing patient age with increasing injury level (*p* = 0.0065). Thus, patients with injuries at level C6/7 had a mean age of 50.4 years, whereas patients with injuries at levels C3/4 and C4/C5 had a mean age of 63.5 years. A highly significant relationship was also found between patient age and injury mechanism. In the cohort, accidents during motorized transportation were predominantly observed in younger patients (*n* = 19, 33.0 years), whereas falls were more frequently the cause of injury in older patients (*n* = 30, 71.5 years).

Preoperatively, 35 patients underwent X-ray as initial diagnostics. Exactly 91 patients received CT imaging. For one patient, externally performed diagnostics were not available. Preoperative MRI was available in 66 of 92 patients (71.7%). MRI was not performed in 26 patients because of emergency surgery, limited availability, contraindications, or according to the treating surgeon’s decision. The combination of CT and MRI was used in 39 patients (42.4%), and diagnosis using X-ray, CT, and MRI was performed in 26 patients (28.2%). In 21 patients (31.8%) who received preoperative MRI, a disc protrusion was observed.

At the centre, 49 patients (53.3%) were treated with anterior plate osteosynthesis after discectomy and insertion of an autologous iliac crest bone graft. Anterior plate osteosyntheses were mainly performed in the period from 2005 to 2015. To maximize primary stability, strict attention was routinely paid to bicortical screw placement in patients treated anteriorly within the plate osteosynthesis. Beginning in 2015, due to improved posterior implants, a paradigm shift occurred so that from then until the end of the observation period, a total of 42 patients (45.6%) were primarily stabilized posteriorly using the respective screw-rod system from DePuy Synthes. Only 1 patient (1.1%) was primarily operated in a combined manner—anteriorly and posteriorly. In this case, due to the high degree of instability of the injury at the cervicothoracic junction as well as the risk of iatrogenic spinal cord compression caused by a combination of hematoma, disc protrusion, and bone fragments, a primary 360 approach was chosen.

**Figure 1:**
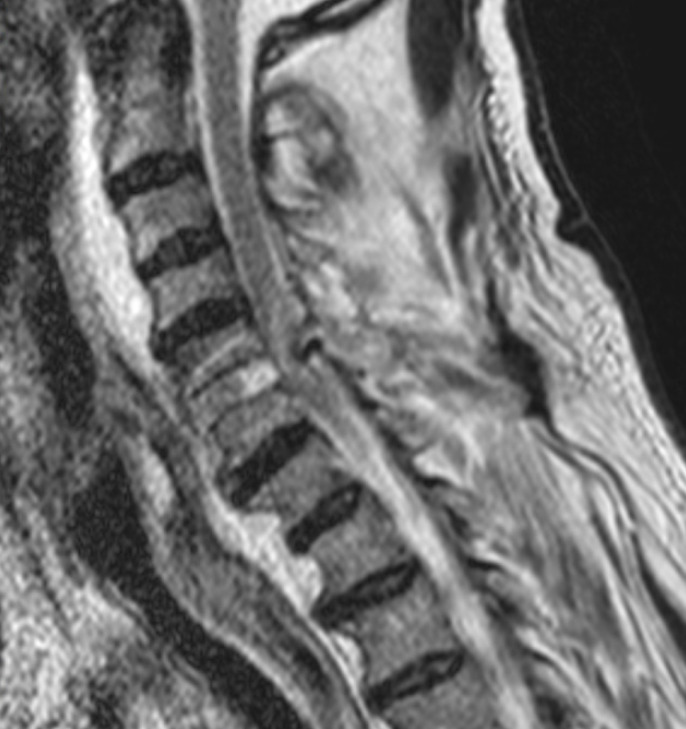
Patient who underwent anterior surgery and experienced postoperative neurological deterioration due to spinal cord compression caused by a disc protrusion and the ligamentum flavum.

**Figure 2:**
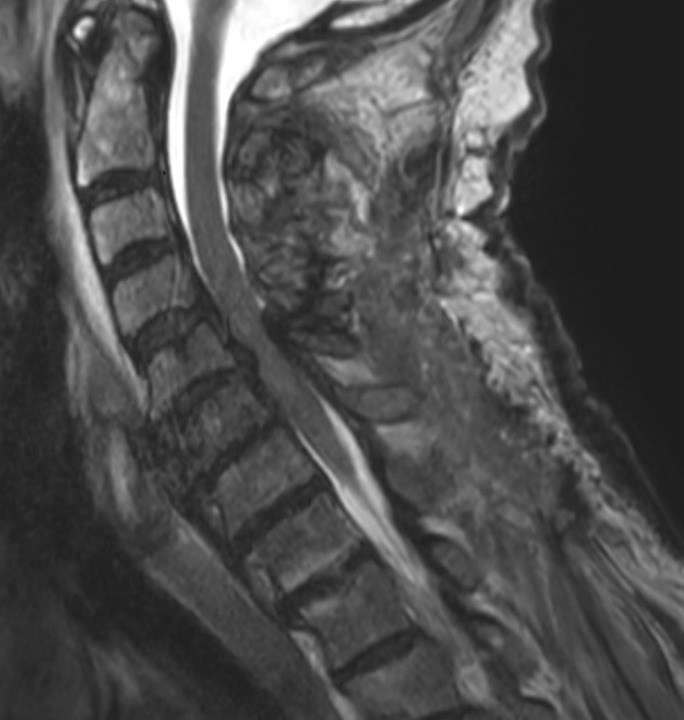
A patient who underwent posterior surgery and experienced postoperative neurological deterioration due to spinal cord compression caused by a disc protrusion and the ligamentum flavum.

A total of 68 patients, corresponding to 73.9%, showed unchanged postoperative ASIA score assessment. In 18 patients (19.6%), improvement occurred. Only 2 patients (2.2%), one anteriorly and one posteriorly treated patient, showed a worse ASIA score postoperatively. It is noteworthy that both patients had MRI-confirmed disc protrusion, although a comparably relevant posterior problem was present in the form of a bulging ligamentum flavum associated with compression of the spinal cord [Figure 1 and Figure 2].

In the subgroup analysis of posteriorly operated patients, improvement in the ASIA score was observed in nine cases (21.4%); in 29 patients (69.0%) the ASIA score remained unchanged, and one patient (2.7%) was assessed postoperatively with a worse ASIA score [Table 3]. In the cohort of anteriorly operated patients, nine patients (18.4%) improved in ASIA score, 38 patients (77.6%) remained stable, and one patient (2.0%) deteriorated [Table 4].

Among the 66 patients who underwent preoperative MRI, traumatic disc protrusion was identified in 21 patients. Of these, six patients showed a radicular deficit at the first neurological assessment. In two patients (9.5%), improvement of the ASIA score occurred clinically. Exactly 18 patients (85.7%), to whom a respective ASIA score had been assigned preoperatively, showed the same ASIA score in the postoperative neurological assessment. One (4.8%) patient with MRI-confirmed protrusion deteriorated neurologically between the initial assessment before surgery and postoperatively [Table 5]. In the group of patients without disc displacement, 12 patients (27.2%) improved. A total of 29 patients (65.9%) were assessed with the same ASIA score, and one (2.3%) patient again showed neurological deterioration [Table 6 and Table 7]. For one patient who underwent an MRI, there was no access to the images taken at the external facility, so the images could not be further interpreted.

With regard to required second operations due to complications defined as primary, significant differences were observed in both main groups. In the group of initially anteriorly operated patients, postoperative radiological instability was demonstrated in six cases, which required subsequent surgery. Two further anteriorly operated patients showed secondary instability but were not revised due to comorbidities, advanced age, and absence of neurological or clinical deterioration. In the posteriorly operated cohort, radiological instability was documented in one patient due to retrospectively technically insufficient treatment; however, this patient was also not reoperated for the reasons mentioned above. Neurological deterioration as a reason for a second operation was shown in one case in each group. In total, nine patients (18.4%) in the anterior group underwent secondary surgery. Of these, seven patients (14.3%) required revision due to a primary complication, while two patients underwent a planned secondary posterior stabilization because of the complex injury pattern and were therefore not classified as primary complications. Only one patient (2.4%) of the posteriorly operated patients required a second operation. With an anteriorly chosen approach, there is a significantly higher probability of requiring a second operation due to radiological deterioration (*p* = 0.021). With regard to neurological deterioration, no significant difference could be demonstrated, with one case in each group (*p* = 0.71).

Subsequently, secondary complications were also defined. These included the occurrence of superficial or deep wound infections, respiratory complications (pneumonia, etc.), implant failure/malposition of screws/plates, implant fracture, hoarseness, or postoperative dysphagia. In both groups, such complications occurred rarely overall: seven patients (14.3%) in the group of anteriorly operated patients compared with nine patients (21.4%) who were stabilized posteriorly. Thus, a slight trend, but no significant difference (*p* = 0.27), could be demonstrated in favour of the anterior group [Table 8].

## 4. Discussion

The optimal surgical strategy for the treatment of unstable injuries of the lower cervical spine remains a subject of controversial discussion despite numerous studies. As also described in the literature, the choice of surgical approach is largely influenced by the neurological status, the injury configuration, the presence of a traumatic disc protrusion, as well as the experience of the surgeon.

In accordance with previous studies, our analysis also shows that both anterior and posterior surgical procedures lead to an overall satisfactory neurological outcome. In both groups, stability of the ASIA score or an improvement could be achieved in the majority of patients, while neurological deterioration occurred rarely and was observed equally in both cohorts. This underlines that both approaches can generally be regarded as safe procedures with respect to neurological outcome.

A central aspect of our investigation was the role of disc protrusion confirmed preoperatively by MRI. In clinical practice, this is often considered an argument in favour of a primarily anterior approach in order to enable direct decompression and to minimize the risk of iatrogenic spinal cord compression. Interestingly, however, no significant difference in neurological outcome was observed in our cohort between patients with and without confirmed disc protrusion. In addition, neurological deterioration occurred independently of the chosen surgical approach. These results support the assumption that disc protrusion per se does not represent a mandatory contraindication for a primarily posterior approach, provided that sufficient reduction can be achieved.

A major difference between the two groups was observed with regard to radiological stability and the necessity of secondary interventions. In the anteriorly operated cohort, the rate of revision surgery was significantly higher, particularly due to postoperative instability. In contrast, in the posteriorly stabilized group, no radiological instability was observed that required renewed surgical treatment. These results suggest a higher primary stability of posterior instrumentation, which may be of clinical relevance, especially in complex injury patterns or involvement of multiple stability columns.

The observed differences in revision rates can also be discussed from a biomechanical perspective. Posterior screw-rod systems primarily stabilize the posterior elements of the cervical spine, particularly the facet joints and posterior ligamentous structures. Through direct reduction and fixation of the facets, effective restoration of segmental stability can be achieved, especially in dislocation-related injuries, in which the posterior structures are predominantly affected.

In contrast, anterior stabilization primarily addresses the anterior and middle columns through discectomy, interbody fusion, and plate osteosynthesis. In cases of pronounced ligamentous injuries or significant involvement of posterior structures, this may not be sufficient to ensure long-term stability. This could explain the increased rate of secondary loss of stability observed in our study after primary anterior treatment.

With regard to secondary complications, no significant difference was observed between the two groups, although a slight trend in favour of the anterior approach was noted. The overall low complication rate in both cohorts underlines the safety of both procedures. Typical approach-specific complications such as dysphagia or hoarseness after anterior surgery, as well as potentially increased soft tissue irritation after posterior procedures, were observed, but had no significant influence on the overall outcome.

An important aspect in the interpretation of our results is the observed temporal change in surgical strategy. While anterior procedures were predominantly used in the earlier observation period, posterior stabilization has increasingly been preferred in recent years. This paradigm shift is likely attributable to advances in implant technology as well as increasing surgical experience with posterior screw systems and represents a potential confounder.

## 5. Limitations

This study has several limitations. It is a retrospective, monocentric analysis with potential selection bias. The assignment to the respective surgical procedure was not randomized but based on clinical decisions that changed over the course of the observation period. A major limitation of this study is the substantial temporal shift in surgical strategy during the study period. Anterior stabilization was predominantly performed in the earlier years of the study, whereas posterior stabilization became the preferred approach in later years. Consequently, surgical approach and treatment era were highly correlated. Because of this near-complete overlap, the independent effects of surgical approach and treatment period could not be reliably separated. Improvements in perioperative management, surgical techniques, implant technology, and postoperative care may have influenced outcomes independently of the surgical approach. Therefore, the present results should be interpreted with caution, and causal inferences regarding the superiority of one surgical approach over the other cannot be drawn from this retrospective cohort.

In addition, not all patients had complete MRI diagnostics, which may limit the assessment of disc pathology. Therefore, findings regarding traumatic disc protrusions should be viewed as preliminary and useful for hypothesis-building rather than as definitive. However, it must be noted that the baseline characteristics were comparable in patients with and without MRI. Additionally, the method used to map Frankel score to ASIA gradings represents a potential limitation of the study. Although Frankel and ASIA grades are broadly comparable, they are not fully equivalent and could therefore lead to a misclassification and compromise the accuracy of neurological outcome assessment.

Adjusted multivariable analyses were not performed because of the limited sample size, the low number of outcome events, and the strong temporal association between surgical approach and treatment period, which would have resulted in unstable estimates and, substantially, multicollinearity. The heterogeneous composition of the injury patterns represents another limitation.

## 6. Conclusions

Both anterior and posterior surgical approaches enabled reliable reduction in unstable lower cervical spine injuries and resulted in comparable neurological outcomes. Posterior stabilization was associated with a significantly lower rate of revision procedures due to postoperative instability, suggesting a tendency towards greater primary mechanical stability, particularly in facet-associated injuries.

Importantly, traumatic disc protrusion neither negatively affected neurological recovery nor was it associated with a measurable benefit from anterior stabilization. These findings challenge the traditional concept of traumatic disc pathology as a dominant determinant of surgical strategy and suggest that disc protrusion alone should not be regarded as an isolated indication for an anterior approach.

Rather, our results support an individualized treatment paradigm in which surgical decision-making is guided by the overall injury pattern, biomechanical considerations, and patient-specific characteristics instead of a single radiographic finding. In this context, the comparable neurological outcomes observed across approaches further support a personalized, pathology-oriented selection of the surgical approach.

Furthermore, both cases of postoperative neurological deterioration were associated with posterior spinal cord compression caused by a bulging ligamentum flavum, pointing to a potentially underrecognized mechanism of secondary neurological injury that warrants further investigation. However, given that this observation is based on only two cases, these findings should be considered purely observational and interpreted with caution until confirmed in larger studies.

While these findings should not be interpreted as a universal treatment recommendation, they provide evidence in favour of a personalized approach to the management of subaxial cervical fracture-dislocations. Prospective studies are needed to validate these observations in larger contemporary cohorts and to further define the role of traumatic disc protrusion within individualized surgical decision-making algorithms.

## Figures and Tables

**Table 1:** Most common types of injuries and neurology at hospitalization.

Type of Injury	*n*	%
Unilateral locked dislocation with unilateral facet fracture	26	28.3
Unilateral perched dislocation with unilateral facet fracture	17	18.5
Bilateral perched dislocation	13	14.1
Bilateral locked dislocation	11	11.9
Unilateral locked dislocation	6	6.5
Bilateral locked dislocation with unilateral facet fracture	6	6.5
Bilateral perched dislocation with bilateral facet fracture	4	4.3
Unilateral locked dislocation with bilateral facet fracture	3	3.3
Other	6	6.6
**Total**	92	100
**ASIA score at hospitalization**	***n***	**%**
ASIA C	31	33.7
Radicular deficit	23	25.0
ASIA E	20	21.7
ASIA D	13	14.1
ASIA B	3	3.3
Other	2	2.2
**Total**	92	100

**Table 2:** Injury level.

Injury Level	*n*	%
**C6/C7**	48	52.2
**C5/C6**	28	30.4
**C4/C5**	7	7.6
**C7/T1**	6	6.5
**C3/C4**	3	3.3
**Total**	92	100

**Table 3:** Change in ASIA score in posterior surgery subgroup (−1 unknown, 0 radicular deficit, ASIA A–E).

Change in ASIA Score		Post Surgery								
Posterior		−1	0	A	B	C	D	E	Other	Total
Hospitalization	−1	0	0	0	0	1	0	0	0	1
	0	0	11	0	0	0	0	1	0	12
	A	0	0	0	0	0	0	0	0	0
	B	0	0	0	1	1	0	0	0	2
	C	2	0	0	0	4	6	0	0	12
	D	0	0	0	0	0	5	1	0	6
	E	0	0	1	0	0	0	8	0	9
	Other	0	0	0	0	0	0	0	0	0
	Total	2	11	1	1	6	11	10	0	42

**Table 4:** Change in ASIA score in anterior surgery group (−1 unknown, 0 radicular deficit, ASIA A–E).

Change in ASIA Score		Post Surgery								
Anterior		−1	0	A	B	C	D	E	Other	Total
Hospitalization	−1	0	0	0	0	0	0	0	0	0
	0	0	11	0	0	0	0	0	0	11
	A	0	0	0	0	0	0	0	0	0
	B	0	0	0	0	1	0	0	0	1
	C	0	0	0	0	10	7	1	0	18
	D	0	0	0	1	0	6	0	0	7
	E	0	0	0	0	0	0	11	0	11
	Other	0	0	0	0	0	0	0	1	1
	Total	0	11	0	1	11	13	12	1	49

**Table 5:** Change in the ASIA score in patients with disc protrusion (−1 unknown, 0 radicular deficit, ASIA A–E).

Change in ASIA Score		Post Surgery								
Protrusion		−1	0	A	B	C	D	E	Other	Total
Hospitalization	−1	0	0	0	0	0	0	0	0	0
	0	0	6	0	0	0	0	0	0	6
	A	0	0	0	0	0	0	0	0	0
	B	0	0	0	0	0	0	0	0	0
	C	0	0	0	0	5	1	1	0	7
	D	0	0	0	0	0	4	0	0	4
	E	0	0	1	0	0	0	3	0	4
	Other	0	0	0	0	0	0	0	0	0
	Total	0	6	1	0	5	5	4	0	21

**Table 6:** Change in the ASIA score in patients without disc protrusion (−1 unknown, 0 radicular deficit, ASIA A–E).

Change in ASIA Score		Post Surgery								
No protrusion		−1	0	A	B	C	D	E	Other	Total
Hospitalization	−1	0	0	0	0	1	0	0	0	1
	0	0	9	0	0	0	0	1	0	10
	A	0	0	0	0	0	0	0	0	0
	B	0	0	0	1	1	0	0	0	2
	C	1	0	0	0	8	9	0	0	18
	D	0	0	0	1	0	4	1	0	6
	E	0	0	0	0	0	0	7	0	7
	Other	0	0	0	0	0	0	0	0	0
	Total	1	9	0	2	10	13	9	0	44

**Table 7:** Overall clinical outcome, by surgical approach and the presence or absence of disc protrusion; CI, confidence interval; OR, odds ratio for neurological improvement vs. deterioration. 95% confidence intervals are reported for improvement and deterioration rates. *p*-values derive from the binomial test comparing improvement and deterioration frequencies. Statistically significant results (*p* < 0.05): Total cohort, anterior, posterior, disc herniation present/absent. Other includes cases with inconsistent documentation.

Subgroup	*n*	Improved *n* (%)	Unchanged *n* (%)	Deteriorated *n* (%)	Unknown *n* (%)	Other *n* (%)	Improvement % (95% CI)	Deterioration % (95% CI)	OR	*p*-Value
**Total cohort**	92	18 (19.6)	68 (73.9)	2 (2.2)	3 (3.3)	1 (1.1)	20.5 (12.6–30.4)	2.3 (0.3–8.0)	11.06	0.0004
**Surgical appr.**										
**Anterior**	49	9 (18.4)	38 (77.6)	1 (2.0)	0 (0)	1 (2.0)	18.8 (8.9–32.6)	2.1 (0.1–11.1)	10.85	0.0215
**Posterior**	42	9 (21.4)	29 (69.0)	1 (2.4)	3 (7.1)	0 (0)	23.1 (11.1–39.3)	2.6 (0.1–13.5)	11.40	0.0215
**Disc hern.**										
**Present**	21	2 (9.5)	18 (85.7)	1 (4.8)	0 (0)	0 (0)	9.5 (1.2–30.4)	4.8 (0.1–23.8)	2.11	1.000
**Absent**	44	12 (27.2)	29 (65.9)	1 (2.3)	2 (4.5)	0 (0)	28.6 (15.7–44.6)	2.4 (0.1–12.6)	16.40	0.0034

**Table 8:** Revision surgeries and complications depending on the surgical approach.

Variable	Subgroup	*n* (%)
**Revision surgery (primary complications)**	Anterior	7 (14.3)
	Posterior	1 (2.4)
**Secondary complications**	Anterior	7 (14.3)
	Posterior	9 (21.4)

## Data Availability

The data presented in this study are available from the corresponding author upon reasonable request. The data are not publicly available due to privacy and ethical restrictions.

## Abbreviations

The following abbreviations are used in this manuscript:

ASIA	American Spinal Injury Association
CT	Computed Tomography
MRI	Magnetic Resonance Imaging
PACS	Picture Archiving and Communication System
ICD	International Classification of Diseases
CI	Confidence interval
OR	Odds ratio

## References

[1] Passias P.G., Poorman G.W., Segreto F.A., Jalai C.M., Horn S.R., Bortz C.A., Vasquez-Montes D., Diebo B.G., Vira S., Bono O.J. (2018). Traumatic Fractures of the Cervical Spine: Analysis of Changes in Incidence, Cause, Concurrent Injuries, and Complications Among 488,262 Patients from 2005 to 2013. World Neurosurg..

[2] Diop M., Epstein D. (2024). A Systematic Review of the Impact of Spinal Cord Injury on Costs and Health-Related Quality of Life. PharmacoEcon. Open.

[3] Futch B.G., Seas A., Ononogbu-Uche F., Khedr S., Kreinbrook J., Shaffrey C.I., Williamson T., Guest J.D., Fehlings M.G., Abd-El-Barr M.M. (2024). Shifting Trends in the Epidemiology of Cervical Spine Injuries: An Analysis of 11,822 Patients from the National Electronic Injury Surveillance System over Two Decades. J. Neurotrauma.

[4] Quarrington R.D., Jones C.F., Tcherveniakov P., Clark J.M., Sandler S.J.I., Lee Y.C., Torabiardakani S., Costi J.J., Freeman B.J. (2018). Traumatic subaxial cervical facet subluxation and dislocation: Epidemiology, radiographic analyses, and risk factors for spinal cord injury. Spine J..

[5] Grant G.A., Mirza S.K., Chapman J.R., Winn H.R., Newell D.W., Jones D.T., Grady M.S. (1999). Risk of early closed reduction in cervical spine subluxation injuries. J. Neurosurg..

[6] Reinhold M., Knop C., Lange U., Rosenberger R., Schmid R., Blauth M. (2006). Reposition von Verrenkungen und Verrenkungsbrüchen der unteren Halswirbelsäule. Unfallchirurg.

[7] Ramakonar H., Fehlings M.G. (2021). ‘Time is Spine’: New evidence supports decompression within 24 h for acute spinal cord injury. Spinal Cord..

[8] Badhiwala J.H., Wilson J.R., Witiw C.D., Harrop J.S., Vaccaro A.R., Aarabi B., Grossman R.G., Geisler F.H., Fehlings M.G. (2021). The influence of timing of surgical decompression for acute spinal cord injury: A pooled analysis of individual patient data. Lancet Neurol..

[9] Kwon B.K., Vaccaro A.R., Grauer J.N., Fisher C.G., Dvorak M.F. (2006). Subaxial cervical spine trauma. J. Am. Acad. Orthop. Surg..

[10] Rizzolo S.J., Piazza M.R., Cotler J.M., Balderston R.A., Schaefer D., Flanders A. (1991). Intervertebral disc injury complicating cervical spine trauma. Spine.

[11] Harrington J.F., Likavec M.J., Smith A.S. (1991). Disc herniation in cervical fracture subluxation. Neurosurgery.

[12] Theodotou C.B., Ghobrial G.M., Middleton A.L., Wang M.Y., Levi A.D. (2019). Anterior Reduction and Fusion of Cervical Facet Dislocations. Neurosurgery.

[13] Reindl R., Ouellet J., Harvey E.J., Berry G., Arlet V. (2006). Anterior reduction for cervical spine dislocation. Spine.

[14] Ren C., Qin R., Wang P., Wang P. (2020). Comparison of anterior and posterior approaches for treatment of traumatic cervical dislocation combined with spinal cord injury: Minimum 10-year follow-up. Sci. Rep..

[15] Nakashima H., Yukawa Y., Ito K., Machino M., El Zahlawy H., Kato F. (2011). Posterior approach for cervical fracture-dislocations with traumatic disc herniation. Eur. Spine J..

[16] Park J.H., Roh S.W., Rhim S.C. (2015). A single-stage posterior approach with open reduction and pedicle screw fixation in subaxial cervical facet dislocations. J. Neurosurg. Spine.

[17] Ernst-Josef Müller G.M. (1997). Wirbelsäulenverletzungen.

